# Comprehensive molecular profiling broadens treatment options for breast cancer patients

**DOI:** 10.1002/cam4.3619

**Published:** 2020-12-04

**Authors:** Hitomi Kawaji, Makoto Kubo, Nami Yamashita, Hidetaka Yamamoto, Masaya Kai, Atsuko Kajihara, Mai Yamada, Kanako Kurata, Kazuhisa Kaneshiro, Yurina Harada, Saori Hayashi, Akiko Shimazaki, Hitomi Mori, Sayuri Akiyoshi, Eiji Oki, Yoshinao Oda, Eishi Baba, Masaki Mori, Masafumi Nakamura

**Affiliations:** ^1^ Department of Surgery and Oncology Graduate School of Medical Sciences Kyushu University Fukuoka Japan; ^2^ Department of Surgery and Science Graduate School of Medical Sciences Kyushu University Fukuoka Japan; ^3^ Department of Anatomic Pathology Graduate School of Medical Sciences Kyushu University Fukuoka Japan; ^4^ Foundation Medicine Business Department Foundation Medicine Unit Chugai Pharmaceutical Co., Ltd. Tokyo Japan; ^5^ Department of Oncology and Social Medicine Graduate School of Medical Sciences Kyushu University Fukuoka Japan

**Keywords:** breast cancer, ERBB2, next generation sequencing, precision oncology, tumor mutational burden

## Abstract

Precision oncology with next generation sequencing (NGS) using tumor tissue with or without blood has begun in Japan. Tumor molecular profiling tests are available, including the OncoGuide™ NCC Oncopanel System and FoundationOne^®^ CDx (F1CDx). Our purpose was to identify potentially actionable genetic alterations in breast cancer with this comprehensive tumor profiling test. We enrolled 115 patients with pathologically diagnosed advanced or metastatic breast cancer. Comprehensive tumor genomic profiling, microsatellite instability, and tumor mutational burden (TMB) were determined using F1CDx. Testing was successful in 109/115 cases (94.8%). Clinically actionable alterations were identified in 76% of advanced breast cancer patients. The most frequent short variants were in *TP53* (48.6%), *PIK3CA* (38.5%), *GATA3* (11.0%), *PTEN* (11.0%), and *BRCA1* (10.1%), and structural variants were in *ERBB2* (24.8%), *MYC* (21.1%), *RAD21* (21.1%), *CCND1* (11.9%), *FGF19* (10.1%), and *PTEN* (10.1%). Regarding human epidermal growth factor receptor (HER)2 status, 106/109 samples (97.2%) were concordant between F1CDx and HER2 testing with immunohistochemistry/fluorescence in situ hybridization. However, *ERBB2* amplification was newly detected in four samples and *ERBB2* mutations were detected in five HER2‐negative breast cancer samples. Oncogenic *BRCA* mutations were found in three samples with F1CDx among 27 germline testing‐negative samples. The mean TMB in all samples was 6.28 mut/Mb and tended to be higher in luminal B and triple‐negative breast cancer (mean = 8.1 and 5.9 mut/Mb, respectively) compared with other subtypes. In conclusion, we established a system for precision oncology and obtained preliminary data with NGS as the first step. The information in this clinical sequencing panel will help guide the development of new treatments for breast cancer patients.

## Graphical abstract

Precision oncology with next generation sequencing (NGS) using tumor tissue with/without blood has begun in Japan. Our purpose was to identify potentially actionable genetic alterations in breast cancer with the comprehensive tumor profiling test, FoundationOne® CDx. Clinically actionable alterations were identified in 76% of advanced breast cancer patients. We have established a system for precision oncology and obtained preliminary data with NGS as the first step.

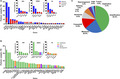

## 1 INTRODUCTION

Cancers are caused by the accumulation of somatic mutations, some of which function as drivers to promote tumorigenesis.^1^, ^2^ Driver mutations that promote tumorigenesis are mainly alterations in three types of genes: oncogenes, tumor suppressor genes, and stability genes that control DNA damage.^3^ Personalized cancer therapy could be achieved by regulating the mutation status of specific molecular drivers in critical signaling pathways. However, the frequency of oncogenic mutations in driver genes varies among tumors and is often affected by ultraviolet light, cigarette smoke exposure, and defects in DNA repair. Oncogenic mutations are less frequent in breast cancer compared with other common solid tumors,^1^, ^4^, ^5^, ^6^ but the distribution of mutational frequency is relatively wider than in other solid tumors and depends on breast cancer subtype. Therefore, genomic profiling for breast cancer needs to be comprehensively investigated because of its diversity.

Several targeted therapies have been demonstrated to be both safe and effective for breast cancer patients, including trastuzumab ^7^ and ado‐trastuzumab emtansine ^8^ for *ERBB2* (which encodes human epidermal growth factor receptor 2 [HER2]‐amplified disease), olaparib^9^ for germline *BRCA*‐mutated disease, pembrolizumab^10^ for microsatellite instability (MSI)‐high disease, and entrectinib^11^ for *NTRK* fusion‐positive disease, as well as hormonal therapies for hormone receptor (HR)‐positive disease. In May 2019, the US Food and Drug Administration (FDA) approved alpelisib in combination with fulvestrant for postmenopausal patients with HR‐positive, HER2‐negative, *PIK3CA*‐mutated, advanced or metastatic breast cancer progressing on or after an endocrine‐based therapy.^12^ Therefore, because effective treatments that target genetic mutations are increasing in the clinic, comprehensive molecular profiling tests for patients with breast cancer are required.

Precision oncology with next generation sequencing (NGS) using tumor tissue with or without blood has been initiated in Japan as of June 2019. Tumor molecular profiling such as using the OncoGuide™ NCC Oncopanel System (NCC Oncopanel)^13^ and FoundationOne^®^ CDx (F1CDx)^14^ are covered by national health insurance for all patients with recurrent and refractory solid tumors including breast cancer. Sunami et al. performed a study using the NCC Oncopanel and found that the percentage of patients with actionable gene aberrations was 59.4% and the access rate to drugs targeting actionable alterations was 13.4%.^13^ However, our understanding of the mechanisms causing somatic mutations in breast cancer and the practice of clinical application using NGS panels are still inadequate.

In this study, we conducted an analysis of 115 breast cancer patients at our institution using F1CDx. Our primary objectives were to investigate the ability of this comprehensive tumor profiling test to identify potentially actionable genetic alterations in breast cancer with the aim of using these systems for precision medicine, and to confirm the success rate of sequencing and diagnostic concordance between F1CDx and existing companion diagnostics (CDx).

## 2 METHODS

### 2.1 Study subjects and study design

**FIGURE 1:**
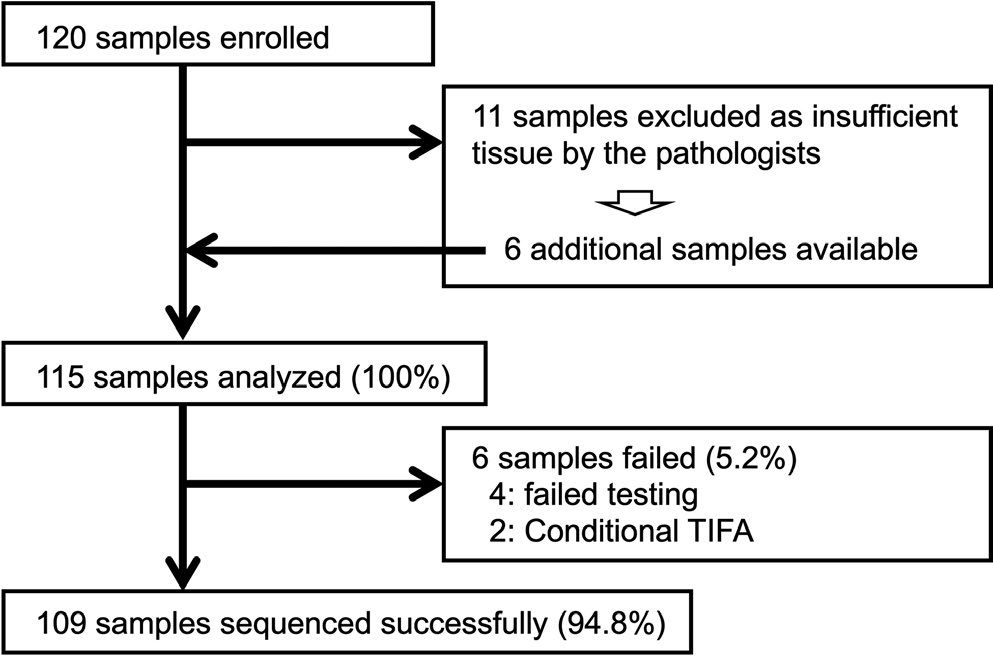
Study flow chart. A total of 120 samples from patients with breast cancer were enrolled; 11 were initially excluded because of insufficient tissue quantity, but additional samples were obtained for 6 of them. Finally, 115 samples were analyzed and sequenced, and genome profiling data were available for 109 of the samples (success rate =94.8%). TIFA, tissue insufficient for analysis.

This study initially included 120 patients with a histologically confirmed diagnosis of breast cancer; 11 were excluded at first because they lacked sufficient tissue for the targeted NGS assay. However, additional samples were obtained for six so only five were eventually excluded (Figure 1). All patients had high‐risk tumors that required neo‐adjuvant or adjuvant chemotherapy, or that were unresectable, and/or recurrent breast cancer. Tumor tissues were collected between January 1, 2015 and March 31, 2019 at Kyushu University Hospital (Fukuoka, Japan). We reviewed patient electronic medical records, pathological information according to the 7th edition of the Union for International Cancer Control staging system,^15^ and genetic results from BRACAnalysis^®^ (Myriad Genetics, Inc.) for olaparib^16^ or the myRisk® multigene panel for hereditary cancer (Myriad Genetics, Inc.).^17^ Informed consent was obtained from all participants included in the study. The study conformed to the principles of the Declaration of Helsinki and was approved by the Institutional Review Board of Kyushu University Hospital (no. 758‐00, 768‐00).

We retrospectively investigated analytical findings of 115 breast cancers with the targeted NGS assay F1CDx. Our primary objectives were the ability of the NGS analysis to detect actionable genetic alterations, the success rate of sequencing, the diagnostic concordance between F1CDx and existing CDx for the same sample, and the associations of genetic alterations and treatments. Our inclusion criteria did not reflect whether patients received experimental or approved agents based on their genetic testing results.

### 2.2 Tumor subtypes and HER2 testing

Tumor subtypes were identified using immunohistochemistry (IHC) staining on tissue acquired by core needle biopsies, excisional biopsies, or surgical resection. All resected specimens used for IHC were fixed in 10% neutral buffered formalin for 6‐72 h. Fixation was performed within 1 h of resection. Estrogen receptor (ER)‐positive or progesterone receptor (PR)‐positive tissues were defined as tumors with ≥1% of tumor cells staining positive for ER or PR. Cancer specimens were defined as HER2‐positive when HER2 IHC staining was scored as 3+ according to standard criteria^18^ or when HER2 gene amplification was detected using fluorescence in situ hybridization (FISH). Cancer specimens were defined as luminal B when the Ki‐67 status was high (>20%) or the PR status was low (<20%) in ER‐positive disease.

### 2.3 Genetic testing with NGS and assessment of genetic test results

We used F1CDx (Foundation Medicine Inc.)^19^ as a targeted multiplex cancer panel test for research purposes only. F1CDx is an NGS‐based *in vitro* diagnostic device for the detection of substitutions, insertion and deletion alterations (indels), and copy number alterations (CNAs) in 309 cancer‐related genes (Table S1A), one promoter region, one noncoding RNA, and select intronic regions from 36 commonly rearranged genes (Table S1B). The assay, therefore, detects alterations in a total of 324 genes. Additionally, genomic signatures are reported, which include MSI and tumor mutational burden (TMB), using DNA isolated from formalin‐fixed paraffin embedded tumor tissue specimens without blood. The F1CDx‐targeted NGS platform has been previously described and validated^14^ and the methods are described briefly here. Samples were prepared according to the manufacturer's instructions as 10 unstained slides (4‐5 µm thick) and one original hematoxylin and eosin staining slide. The tumor size was required to be more than 1 mm^3^. The optimal percentage of tumor nuclei was 30% or more, and a minimum of 20% was required. The clinical physician chose the sample for testing, then, pathologists assessed sample suitability and prepared the slides. If the sample was judged to be inappropriate by the pathologists, more sample was added or another sample was chosen for the test.

To determine the MSI status, 95 intronic homopolymer repeat loci (10‐20 bp long in the human reference genome) with adequate coverage on the F1CDx assay were analyzed for length variability and compiled into an overall MSI score via principal components analysis.^20^ Each sample was assigned a qualitative status of MSI‐High (MSI‐H) or MSI‐Stable (MSS), or a low coverage (<250× median) status of MSI‐unknown.^20^ TMB by F1CDx was defined by counting the total number of all synonymous and nonsynonymous variants present at ≥5% allele frequency (after filtering) and was reported as mutations per megabase (mut/Mb) rounded to the nearest integer.

### 2.4 Reporting and annotation of genetic testing results

The sequencing test, data analysis, and annotation were conducted by Foundation Medicine Inc. The final report in F1CDx includes any detected genomic findings and FDA‐approved therapeutic options, such as anti‐HER2 therapies (Herceptin^®^ [trastuzumab], Kadcyla^®^ [ado‐trastuzumab emtansine], and Perjeta^®^ [pertuzumab]), Keytruda^®^ (pembrolizumab), or Rozlytrek^®^ (entrectinib) for CDx‐associated findings of *ERBB2* amplification, MSI‐High, or *NTRK* gene fusions in breast cancer, respectively. Complete lists of the 309 and 36 genes assayed for the detection of base substitutions, insertion/deletions, CNAs, and select rearrangements are shown in Table S1A and B, respectively. Final single nucleotide variant (SNV) calls were made at a mutant allele frequency (MAF) ≥ 5% (MAF ≥ 1% at hotspots) with filtering for strand bias, read location bias, and the presence of two or more controls. Additionally, information regarding clinical trials was provided. The criteria for inclusion of genetic alterations in the final report available to the clinician have been described previously^19^, ^21^ and are briefly summarized here. For base substitutions, final calls were made at a MAF ≥5% or ≥1% for known mutation hotspots after filtering for read location bias and strand bias. For CNAs, focal amplifications were called at six or more copies and homozygous deletions were called at zero copies. Gene fusions were detected by assessing chimeric read pairs, and the function of the rearrangements was predicted. Because the final report that the clinician used to make clinical decisions did not contain MAFs or CNA data at this time, this information was not included in the study. Moreover, variants of unknown significance (VUS) were described in the final report but are not discussed in this study.

### 2.5 Statistical analysis

The sample size was determined by the number of patients available during the duration of research until the beginning of public insurance. Therefore, no formal statistical hypotheses were assessed. Logistic regression was used to compare continuous variables and χ^2^ tests were used to compare categorical variables between groups in TMB. Values of *p* < 0.05 were considered statistically significant. Statistical analysis was carried out using JMP 11 software (SAS Institute Inc.) and Graph Pad Prism version 8.0 (Graph Pad, Inc.).

## 3 RESULTS

### 3.1 Patient characteristics

A total of 115 breast cancer samples (all from female patients) were tested using F1CDx. Sequence tests of 109 samples were successful while testing in six samples failed (Table S2A). The clinicopathologic information of patients whose testing failed is listed in Table S2B. Of the six samples that failed, five samples had a small tumor volume.

The clinical and pathological characteristics of patients with successful testing results are summarized in Table 1. The median patient age was 62 years and 14 patients were aged under 39 years. The distribution of patients among clinical subtypes was as follows: luminal B‐like disease, 43 cases (39.4%); triple‐negative breast cancer (TNBC), 34 cases (31.2%); HER2‐positive disease, 24 cases (22%, including 13 luminal‐HER2 cases); and luminal A‐like disease, 8 cases (7.3%). Most patients had clinical stage IV or recurrent tumors (54.1%), and the others had stage III (26.6%) and II (19.3%) with tumors resected at a high risk of recurrence.

**TABLE 1:** Patients’ clinicopathologic characteristics.

Variable	Classification	Result, *n* (%)
Age, years	Median	62
	Range	22–92
Sex	Female	109 (100.0)
	Male	0 (0.0)
Subtype	Luminal A	8 (7.3)
	Luminal B	43 (39.4)
	Luminal‐HER2	13 (11.9)
	HER2	11 (10.1)
	Triple‐negative	34 (31.2)
Stage	II	21 (19.3)
	III	29 (26.6)
	IV	59 (54.1)
Sample type	Primary tumor	74 (67.9)
	Metastatic tumor	35 (32.1)
Sample method	Biopsy	61 (56.0)
	Resection	48 (44.0)
Specimen site	Breast	82 (75.2)
	Lymph node	6 (5.5)
	Skin	4 (3.7)
	Chest wall	4 (3.7)
	Liver	4 (3.7)
	Lung	2 (1.8)
	Abdominal wall	2 (1.8)
	Ovary	2 (1.8)
	Colon	1 (0.9)
	Thymus	1 (0.9)
	Brain	1 (0.9)

Of the 109 breast cancer samples, 74 (67.9%) were tested from the primary tumor and 35 (32.1%) were from metastatic sites. Sixty‐one samples (56.0%) were tested from biopsy specimens and 48 (44.0%) were tested from resected samples. The most common specimen site was the breast (75.2%), and the next most common site was the lymph node (5.5%).

### 3.2 Genetic variants detected by F1CDx

**FIGURE 2:**
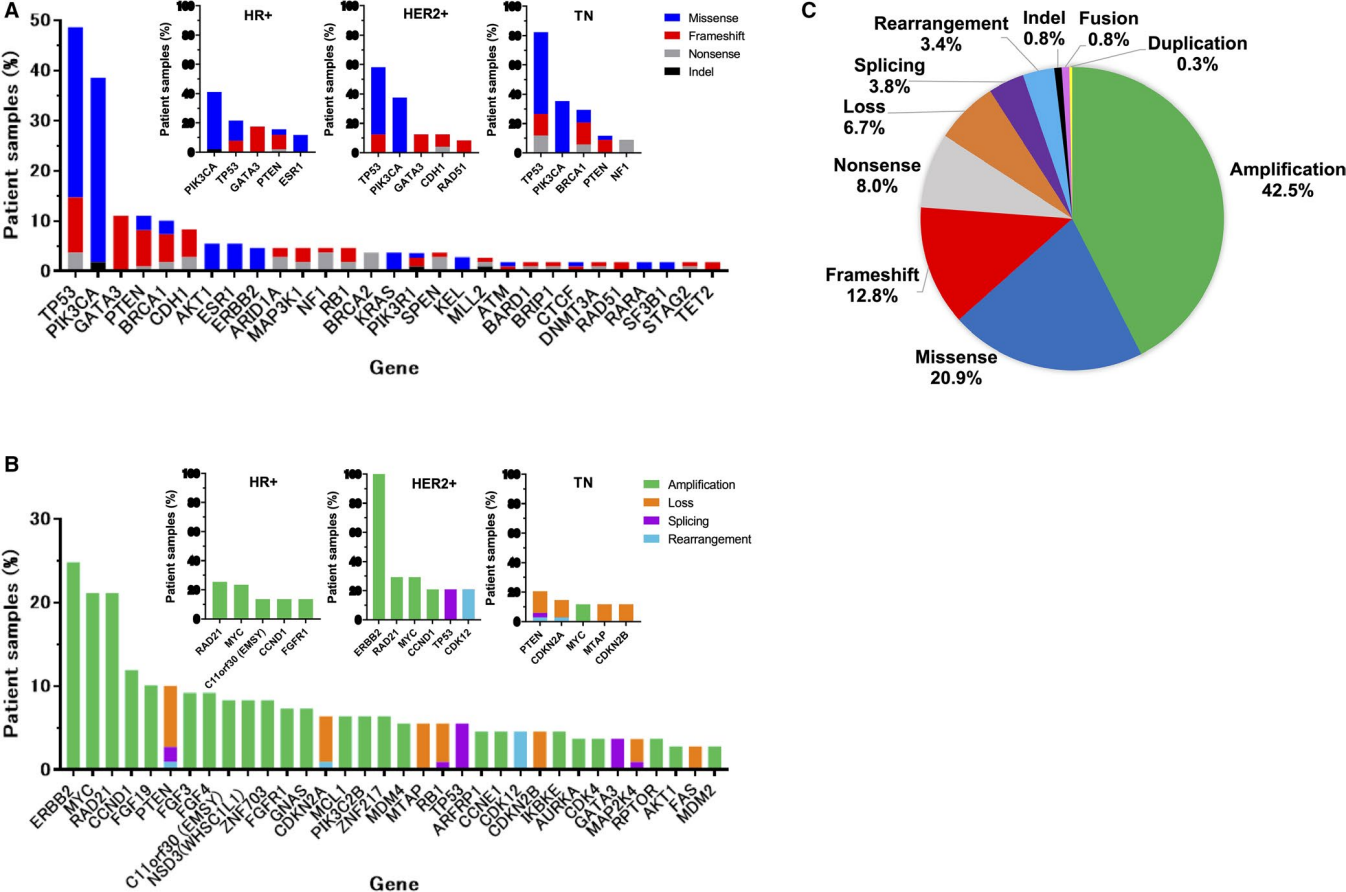
Potentially actionable alterations in patient samples. Frequency of short variants (SNV/InDel) (A) and structural variants (CNA/fusion/loss/splicing/rearrangement) (B) in most commonly altered genes. C, Proportion of clinically actionable alterations.

Among the 109 samples, 108 showed at least one genetic variant with F1CDx; in one sample, no variant was detected. The frequency of the variants is shown in Figure 2. Short variants (single nucleotide variant and indel) were most common in *TP53* (48.6%), *PIK3CA* (38.5%), *GATA3* (11.0%), *PTEN* (11.0%), and *BRCA1* (10.1%; Figure 2A). Of the short variants, missense mutations were mainly found in *TP53* and *PIK3CA*, and frameshift mutations were frequent in *GATA3*, *PTEN*, *BRCA1*, and *CDH1*. *PIK3CA* in HR‐positive disease and *TP53* in HER2‐positive and TNBC were the most frequent short variants (Figure 2A). The most frequent structural variants (such as CNA or fusion and loss) were in *ERBB2* (24.8%), *MYC* (21.1%), *RAD21* (21.1%), *CCND1* (11.9%), *FGF19* (10.1%), and *PTEN* (10.1%; Figure 2B). These structural variants were all detected as amplifications. TNBCs showed losses in *PTEN*, *CDKN2A*, *MTAP*, and *CNKN2B* (Figure 2B). The most frequent alteration was amplifications (42.5%), followed by missense mutations (20.9%), frameshifts (12.8%), nonsense mutations (8.0%), and losses (8.0%; Figure 2C).

### 3.3 Proportion of alterations annotated based on clinical actionability

**FIGURE 3:**
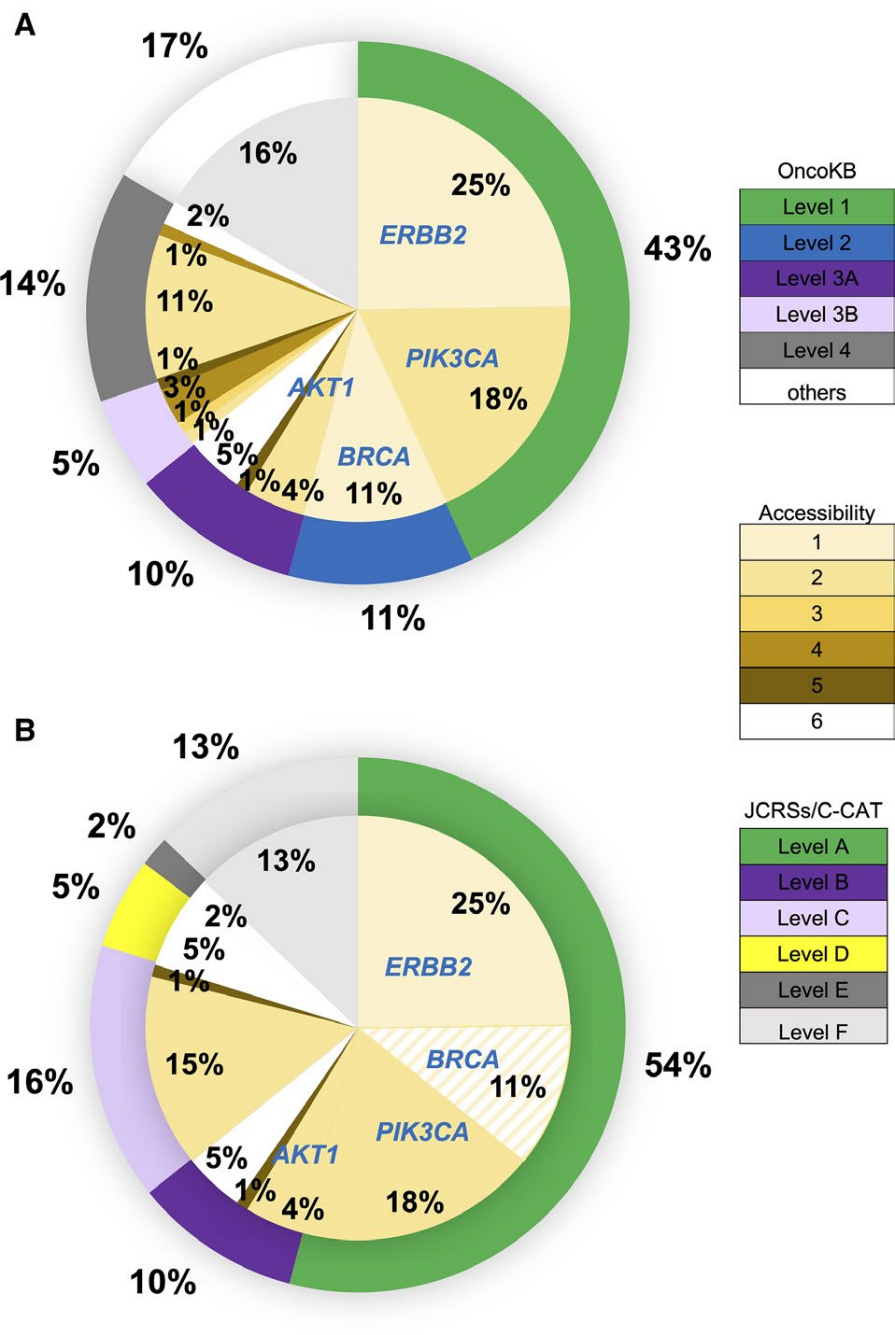
Proportion of alterations annotated on the basis of their clinical actionability according to OncoKB and the consensus of three major Japanese cancer‐related societies. The outer pie chart demonstrates the proportion of the levels of evidence according to OncoKB (A) and JCRSs/C‐CAT (B). The inner pie chart indicates the proportion of accessibility to treatments in Japan according to JCRSs/C‐CAT. Figures show distribution (%). JCRSs, three major Japanese cancer‐related societies; C‐CAT, Center for Genomics and Advanced Therapeutics

We next assessed whether variants detected using F1CDx indicated the use of drugs and treatments based on medical evidence. First, the samples were classified into five levels according to OncoKB clinical evidence levels ^7^ (Table S3A), as shown in the outer pie chart in Figure 3A. Because some FDA‐approved drugs are not permitted in Japan, the samples were then classified with the clinical evidence level according to the consensus of three major Japanese cancer‐related societies and the Center for Cancer Genomics and Advanced Therapeutics (C‐CAT; Table S3B) and accessibility (Table S3C), as shown in the inner pie chart in Figure 3B. The final reports to clinicians are summarized in Table S3D.

### 3.4 Concordance of *ERBB2* results with HER2 testing and F1CDx

IHC staining was generally used to classify HER2 status, but in cases where the HER2 score was 2+ samples were then tested by FISH. The comparison of existing HER2 test results and F1CDx is shown in Table 2A. Three samples were newly detected with *ERBB2* amplifications and five samples were detected with *ERBB2* mutations. One sample had both *ERBB2* amplification and mutation. Of the 109 samples, 106 (97.2%) were concordant in the classification of HER2 status. Table 2B lists details of the eight samples with newly detected *ERBB2* amplifications and oncogenic variants. Five samples with *ERBB2* mutations were reported with HER2‐negative disease.

**TABLE 2:** Comparison of HER2 testing and F1CDx analyses for *ERBB*. A, Concordance between HER2 testing and F1CDx results for *ERBB2*; (B) Conversion and oncogenic variants in *ERBB2* with F1CDx

(A) HER2 testing		F1CDx (*ERBB2*)				Total
IHC	FISH	Amplification	Low amplification^a^	Mutation	ND	
3+		21	0	0	0	21
2+	Positive	1	2	0	0	3
	Negative	0	0	2	22	24
1+		1^b^	1	3^b^	30	35
0		1	0	0	26	27
	Total	24	3	5	78	110

(B) Sample no	HER2 testing		F1CDx
	IHC	FISH	*ERBB2*
064	2+	Negative	S563C
070	2+	Negative	R896H
035	1+		D769Y
100	1+		G776V
073	1+		Low amplification^a^
079	1+	Positive	Amplification/L755S
022	0 (partially 3+)		Amplification

Abbreviations: F1CDx, FoundationOne^®^ CDx, FISH, fluorescence in situ hybridization, HER2, human epidermal receptor type 2; IHC, immunohistochemical staining.

^a^ Low amplification: *ERBB2* amplification of copy number 4 was detected.

^b^ One patient showed both *ERBB2* amplification and mutation.

### 3.5 Concordance of *BRCA1* and *BRCA2* results with BRACAnalysis and F1CDx

Thirty‐five patients were tested by germline genetic testing. We compared the results of oncogenic variants identified in *BRCA1*/*2* from germline testing such as BRACAnalysis or myRisk with F1CDx somatic testing (Table 3A). Seven samples diagnosed as positive by germline testing were also judged as positive at the same single site using F1CDx. *BRCA* pathogenic mutations as a secondary finding were suspected in two patients (no. 028 and 091). In one sample (No. 097) diagnosed as positive by myRisk, the same mutation was identified as VUS by F1CDx. Although three samples were negative by germline *BRCA* testing, these samples showed oncogenic mutations using F1CDx. The genetic results of germline and somatic oncogenic variants in *BRCA1*/*2* identified using germline genetic testing and F1CDx are shown in Table 3B.

**TABLE 3:** Comparison of germline and somatic mutations in *BRCA1*/*2*. A, Summary; (B) detailed results of cases

(A) Germline genetic testing^a^	F1CDx		Total
Oncogenic variant	Oncogenic variant	Not detected	
Positive	7	1^b^	8
Negative	3	24	27
Not examined	7	67	74
Total	17	92	109

(B) Sample no	Subtype	Germline genetic testing^#1^		F1CDx	
		*BRCA1*	*BRCA2*	*BRCA1*	*BRCA2*
002	TNBC	G1738R		G1738R	—
007	TNBC	2050delG		C644fs*7	—
026	TNBC	1135insA		V340fs*6	—
028	TNBC	K893fs*106		K893fs*106	—
097	TNBC	C64R		C64R^c^	—
099	TNBC	Positive		R1085*	—
091	HER2	81‐1G>A		splice site 81‐1G>A	—
201	Luminal B		S547X	—	S547*
004	TNBC	—	—	G275D	—
035	TNBC	—	—	E1011*	—
088	Luminal B	—	—	—	E2474*
029	TNBC	NE	NE	S1374fs*1	—
030	TNBC	NE	NE	truncation exon 10	—
060	TNBC	NE	NE	I1159fs*50	—
040	Luminal B	NE	NE	E111fs*3	—
055	Luminal B	NE	NE	—	R2318*
216	Luminal B	NE	NE	—	R2318*
074	Luminal‐HER2	NE	NE	—	truncation intron 7

Abbreviations: F1CDx, FoundationOne® CDx; HER2, human epidermal receptor type 2; NE, not examined; TNBC, triple‐negative breast cancer.

^a^ Germline genetic testing included BRACAnalysis and multigene panel testing, myRisk.

^b^ Variant of unknown significance.

^c^ This mutation was found as a variant of unknown of significance in the F1CDx report.

### 3.6 MSI and TMB

The results of MSI and TMB are shown in Table 4. Only one case was identified as MSI‐high and was diagnosed with Lynch syndrome. The microsatellite status of 107 samples was stable although the status of two samples could not be determined. The TMB level of the patient with the MSI‐high tumor was the maximum of 82 mut/Mb.

**TABLE 4:** A, Microsatellite instability (MSI) with F1CDx; (B) Tumor mutation burden (TMB) with F1CDx

(A) MSI status	*n* (%)
MSS	107 (98.2)
MSI‐high	1 (0.9)
Cannot be determined	1 (0.9)

(B) TMB (mut/Mb)	Classification	*n* (%)
>20	High	3 (2.8)
6‐20	Intermediate	35 (32.1)
0‐5	Low	69 (63.3)
Cannot be determined		2 (1.8)

Abbreviations: MSS, microsatellite instability stable; mut/Mb, mutations/megabase.

**FIGURE 4:**
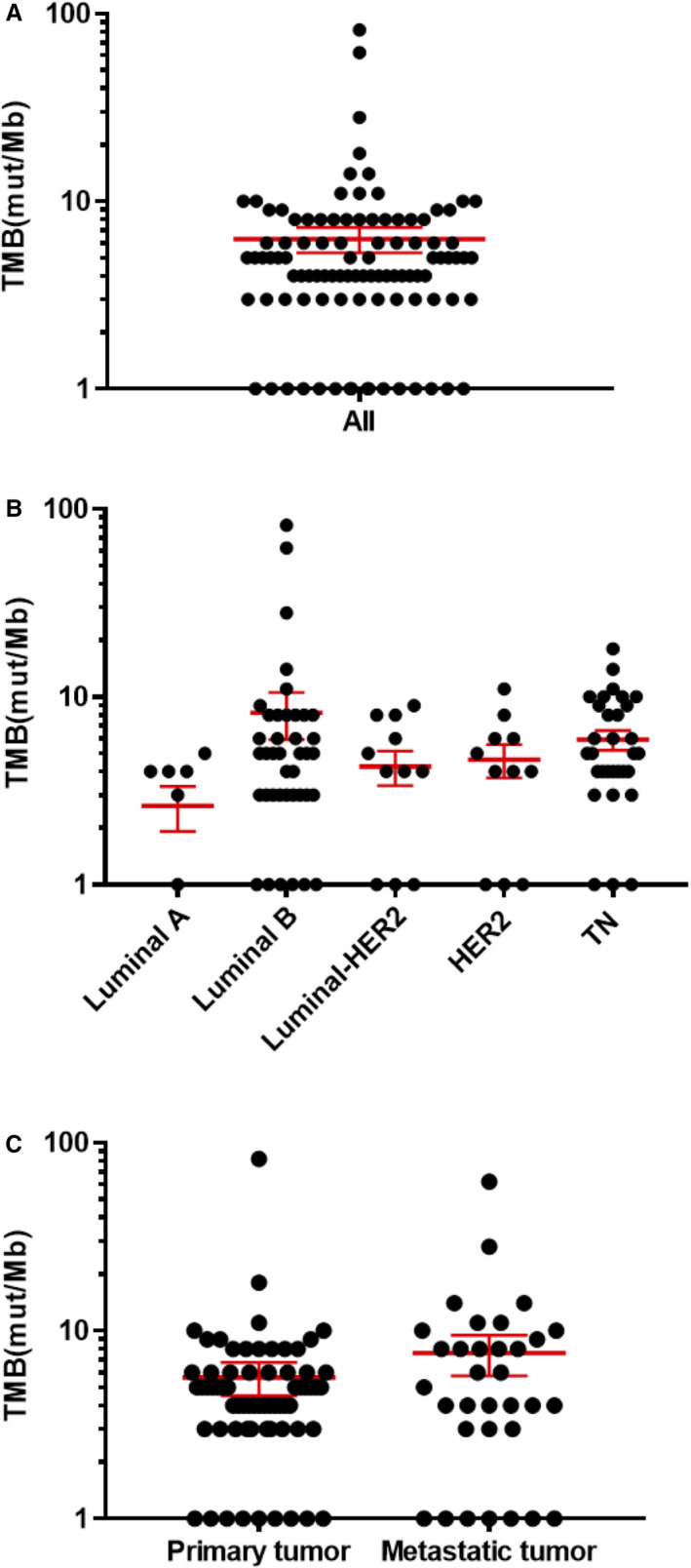
Tumor mutation burden (TMB). Dots represent the TMB of samples; red horizontal bars represent the mean and error bars show ±standard error of the mean (SEM). The vertical axis (log‐scaled) shows the TMB (mut/Mb) in all samples (A), by subtype (B), and by tumor type (C). mut/Mb, mutations/megabase.

In F1CDx reports, TMB was classified into three categories: high, intermediate, and low. Three samples were classified as high, 33 as intermediate, and 71 as low. The analyses of TMB are shown in Figure 4. The mean TMB in all samples was 6.28 ± 0.97 mut/Mb (mean ± SEM; Figure 4A). There were no significant differences in groups by subtypes (Figure 4B) or sample sites (Figure 4C). We observed the trend that TMB was higher in luminal B and TNBC (mean = 8.1 and 5.9 mut/Mb, respectively) compared with other subtypes.

## 4 DISCUSSION

Over the last decade, the evolution of NGS technology and innovation in bioinformatics have increased the ability of large‐scale tumor sequencing panels to target the full coding region of hundreds of cancer‐related genes.^14^, ^22^, ^23^ Recently, however, tumor‐only analysis has been the typical clinical standard.^24^ In the present study, we retrospectively reviewed our experience of the first 115 breast cancer patients undergoing comprehensive molecular sequencing tests with F1CDx as a preclinical setting.

Our results showed that 109 (94.8%) of the 115 samples were successful for sequence testing in cooperation with pathologists. The current success rate (94.8%) as the primary endpoint was relatively higher than that of MSK‐IMPACT (341‐410 genes; Memorial Sloan Kettering Cancer Center; 86.4%, 10,945/12,670 samples),^25^ OncoPrime (215 genes) at Kyoto University (Kyoto, Japan; 91.9%, 57/62 formalin‐fixed paraffin embedded samples),^26^ and NCC Oncopanel (114 genes) at the National Cancer Center Hospital (Tokyo, Japan; 88.2%, 187/212 specimens).^13^

There are few reports about genetic alternations identified using F1CDx and their association with clinicopathologic characteristics in breast cancer. Some studies showed the alternations of many tumor types including breast cancer, but did not necessarily focus on it alone.^14^, ^21^ Recently, Freitag et al. reported genetic insights into the biology of advanced breast cancers and summarized the most frequent clinically actionable genetic alterations identified using F1CDx in a cohort of 223 advanced breast cancers.^27^ The most frequent variants were in *TP53* (54%), *PIK3CA* (35%), *MYC* (22%), *CCND1* (20%), and *FGF19* (20%), consistent with previous studies as well as our results in the Japanese cohort. However, a more detailed comparison of alternation differences among races or regions is needed.

*ERBB2* encodes the HER2 receptor tyrosine kinase. *ERBB2* mutations, such as those identified in this study, have been shown to be activating.^28^ The HER2 status of 109 (96.3%) of the 115 samples in this study was concordant between F1CDx, FISH, and IHC findings. However, *ERBB2* amplification was newly detected in four samples (3.7%) and *ERBB2* mutations were detected in five samples (4.6%) in HER2‐negative breast cancer. Frampton et al. reported that sensitivity with F1CDx was >95% for CNAs and that more than 40% of all *ERBB2* alterations were point mutations or indels in nonamplified specimens.^14^ Wang et al found that *HER2* somatic mutations occurred at a higher frequency in HER2‐negative breast cancer, and that patients with these mutations had poor survival.^29^ Additionally, Connell et al. revealed that the presence of HER2 mutations in breast cancer (1.8%) negatively correlated with overall survival using TCGA data.^30^ *ERBB2* activating mutations have predicted clinical responses to regimens that include ERBB2‐targeted therapies, such as trastuzumab,^31^ lapatinib,^32^ and neratinib.^33^ Therefore, these additional findings would enable the use of HER2‐targeted therapies in newly discovered patients with ERBB2 amplification or activating mutations.

Tumors with alterations that inactivate BRCA1/2 may confer sensitivity to poly (ADP‐ribose) polymerase (PARP) inhibitors such as olaparib,^9^ rucaparib,^34^ or niraparib,^35^ and to DNA‐damaging drugs such as cisplatin and carboplatin. In the TNT—the “Triple Negative Trial”—which is a randomized phase III trial of carboplatin compared to docetaxel for patients with metastatic or recurrent locally advanced TNBC, TNBC patients with germline *BRCA1*/*2* mutations had a significantly better response to carboplatin than to docetaxel, but not significantly better than in patients with tumor *BRCA1*/*2* mutations.^36^ Clinical responses to PARP inhibitors have been reported for ovarian cancer patients with deleterious or suspected deleterious germline or somatic *BRCA1*/*2* mutations,^37^, ^38^ so evaluation of *BRCA* oncogenic mutations using F1CDx has become a companion diagnostic for ovarian cancer patients.^13^ In this study, *BRCA* oncogenic mutations were found in three samples with F1CDx despite negative results with BRACAnalysis. The distribution of germline and somatic oncogenic variants detected upon tumor analysis was 4:1–5:2.^39^, ^40^ Therefore, further research is needed to determine whether DNA‐damaging drugs have a better response for breast cancer with somatic *BRCA1*/*2* mutations.

MSI is a condition of genetic hypermutability that is targeted by immune checkpoint inhibitors in various solid tumors.^41^ The MSI‐H/MSS defined by F1CDx is based on a genome‐wide analysis of 95 intronic homopolymer repeat loci with adequate coverage, not based on the five or seven MSI loci described in current clinical practice guidelines. For each sample the repeat length is calculated in each read that spans each of the 95 loci, which produces an MSI score that is designated MSI‐H or MSS by manual unsupervised clustering.^20^ In this study, microsatellite status was high in only one patient diagnosed with Lynch syndrome. The frequency of MSI in breast cancer is uncommon, at approximately 1%.^42^, ^43^ Therefore, comprehensive genomic profiling is useful to simultaneously investigate infrequent biomarkers.

TMB defined by F1CDx is based on counting the total number of all synonymous and nonsynonymous variants present at an allele frequency of ≥5%. However, the clinical validity of TMB defined by this panel has not been established. The results of the CheckMate 227 (NCT02477826) trial validated the benefit of immune checkpoint inhibitors in lung cancer and the role of high TMB (≥10 mut/Mb) determined by F1CDx as a biomarker for patient selection.^44^ Yarchoan et al reported a strong relationship between the TMB validated with F1CDx and the activity of anti‐PD‐1 therapies across multiple cancers.^45^ In this study, the TMB level of the luminal B‐like patient with an MSI‐high tumor was the maximum of 82 mut/Mb and TMB‐high (≥10 mut/Mb) tumors were 12.0% (n = 13). The mean TMB in all samples was 6.28 mut/Mb, and TMB tended to be higher in luminal B and TNBC patients (mean = 8.1 and 5.9 mut/Mb, respectively) compared with patients of other subtypes. Karn et al reported that the average total mutation count in breast cancer was highest in TNBC, followed by HER2 type, luminal B, and luminal A.^46^ There was no characteristic difference in genetic alterations or tumor mutation burden between samples from patients receiving treatment and treatment‐naïve patients (data not shown). The average tumor mutational load in breast cancer is not particularly high compared with other solid tumors,^1^, ^4^, ^5^, ^6^ but the range of TMB is wider in breast cancer than in other solid tumors and varies according to subtypes. Therefore, an analysis of TMB may enable new treatment options to be offered to breast cancer patients. On June 16, 2020, the FDA approved the F1CDx assay as a companion diagnostic to pembrolizumab for adult and pediatric patients with TMB‐high unresectable or metastatic solid tumor.^47^

This is the first report to compare widely comprehensive tumor molecular profiling testing achieved using F1CDx with existing CDx results for breast cancer patients. We had preliminary data for these patients and this study established a system for precision oncology, which included the F1CDx profiling test, a panel of consulting experts, and a system to annotate results for breast cancer patients. This study had several limitations. First, samples tested with F1CDx included both the primary and metastatic sites and only retrospectively collected cases. Second, we were unable to use potentially actionable alterations for patient treatments because we used F1CDx as a targeted multiplex cancer panel test only for research purposes. Our final goals are to identify biomarkers that are functional driver mutations and offer safer and more efficacious treatments for breast cancer patients.

## 5 CONCLUSION

The present study revealed that tumor genetic profiling using a targeted NGS panel F1CDx identified actionable alterations in 76% of patients with advanced breast cancer. This clinical sequencing panel could add new essential information to guide daily medical care for breast cancer patients. Therefore, the comprehensive molecular profiling is likely to broaden treatment options and provide clinical benefit to many patients with breast cancer.

## CONFLICT OF INTEREST

MK has received honoraria as a speaker or in a consultant/advisory role from Chugai Pharmaceutical Co. (Tokyo, Japan). EB has received honoraria and a research grant from Chugai Pharmaceutical Co. The other authors declare no conflicts of interest.

## AUTHOR CONTRIBUTIONS

HK and MK (Makoto Kubo) contributed equally to this work. MK (Makoto Kubo) and AK designed the research; HM, MK (Makoto Kubo), NY, MK (Masaya Kai), MY, KK (Kanako Kurata), KK (Kazuhisa Kaneshiro), YH, SH, AS, HM, SA, and EO performed the research; HY and YO provided the clinical samples which were diagnosed pathologically; HM and MK (Makoto Kubo) analyzed the data, and wrote the paper; EB, MM, and MN is the supervision.

## Supporting information

Supplementary Material

## Data Availability

Author elects to not share data: Research data are not shared.
