# Supplementary material for: Comprehensive molecular profiling broadens treatment options for breast cancer patients
Source: Cancer Med. 2020 Dec 4;10(2):529–39. doi: 10.1002/cam4.3619 (PMC7877356; doi:10.1002/cam4.3619)
Supplement: Supplementary file 1 — Supplementary Material [file CAM4-10-529-s001.doc]

**Supplementary Tables**

**Table S1.** List of genes analyzed with F1CDx

A. DNA gene list: the entire coding sequence was examined for the detection of base substitutions, insertion/deletions, and copy number alterations.

| *ABL1* | *ACVR1B* | *AKT1* | *AKT2* | *AKT3* | *ALK* | *ALOX12B* | *AMER1* | *APC* | *AR* |
| --- | --- | --- | --- | --- | --- | --- | --- | --- | --- |
| *ARAF* | *ARFRP1* | *ARID1A* | *ASXL1* | *ATM* | *ATR* | *ATRX* | *AURKA* | *AURKB* | *AXIN1* |
| *AXL* | *BAP1* | *BARD1* | *BCL2* | *BCL2L1* | *BCL2L2* | *BCL6* | *BCOR* | *BCORL1* | *BRAF* |
| *BRCA1* | *BRCA2* | *BRD4* | *BRIP1* | *BTG1* | *BTG2* | *BTK* | *C11orf30* | *CALR* | *CARD11* |
| *CASP8* | *CBFB* | *CBL* | *CCND1* | *CCND2* | *CCND3* | *CCNE1* | *CD22* | *CD274* | *CD70* |
| *CD79A* | *CD79B* | *CDC73* | *CDH1* | *CDK12* | *CDK4* | *CDK6* | *CDK8* | *CDKN1A* | *CDKN1B* |
| *CDKN2A* | *CDKN2B* | *CDKN2C* | *CEBPA* | *CHEK1* | *CHEK2* | *CIC* | *CREBBP* | *CRKL* | *CSF1R* |
| *CSF3R* | *CTCF* | *CTNNA1* | *CTNNB1* | *CUL3* | *CUL4A* | *CXCR4* | *CYP17A1* | *DAXX* | *DDR1* |
| *DDR2* | *DIS3* | *DNMT3A* | *DOT1L* | *EED* | *EGFR* | *EP300* | *EPHA3* | *EPHB1* | *EPHB4* |
| *ERBB2* | *ERBB3* | *ERBB4* | *ERCC4* | *ERG* | *ERRFI1* | *ESR1* | *EZH2* | *FAM46C* | *FANCA* |
| *FANCC* | *FANCG* | *FANCL* | *FAS* | *FBXW7* | *FGF10* | *FGF12* | *FGF14* | *FGF19* | *FGF23* |
| *FGF3* | *FGF4* | *FGF6* | *FGFR1* | *FGFR2* | *FGFR3* | *FGFR4* | *FH* | *FLCN* | *FLT1* |
| *FLT3* | *FOXL2* | *FUBP1* | *GABRA6* | *GATA3* | *GATA4* | *GATA6* | *GID4 (C17orf39)* | *GNA11* | *GNA13* |
| *GNAQ* | *GNAS* | *GRM3* | *GSK3B* | *H3F3A* | *HDAC1* | *HGF* | *HNF1A* | *HRAS* | *HSD3B1* |
| *ID3* | *IDH1* | *IDH2* | *IGF1R* | *IKBKE* | *IKZF1* | *INPP4B* | *IRF2* | *IRF4* | *IRS2* |
| *JAK1* | *JAK2* | *JAK3* | *JUN* | *KDM5A* | *KDM5C* | *KDM6A* | *KDR* | *KEAP1* | *KEL* |
| *KIT* | *KLHL6* | *KMT2A (MLL)* | *KMT2D (MLL2)* | *KRAS* | *LTK* | *LYN* | *MAF* | *MAP2K1* | *MAP2K2* |
| *MAP2K4* | *MAP3K1* | *MAP3K13* | *MAPK1* | *MCL1* | *MDM2* | *MDM4* | *MED12* | *MEF2B* | *MEN1* |
| *MERTK* | *MET* | *MITF* | *MKNK1* | *MLH1* | *MPL* | *MRE11A* | *MSH2* | *MSH3* | *MSH6* |
| *MST1R* | *MTAP* | *MTOR* | *MUTYH* | *MYC* | *MYCL* | *MYCN* | *MYD88* | *NBN* | *NF1* |
| *NF2* | *NFE2L2* | *NFKBIA* | *NKX2-1* | *NOTCH1* | *NOTCH2* | *NOTCH3* | *NPM1* | *NRAS* | *NT5C2* |
| *NTRK1* | *NTRK2* | *NTRK3* | *P2RY8* | *PALB2* | *PARK2* | *PARP1* | *PARP2* | *PARP3* | *PAX5* |
| *PBRM1* | *PDCD1* | *PDCD1L G2* | *PDGFRA* | *PDGFRB* | *PDK1* | *PIK3C2B* | *PIK3C2G* | *PIK3CA* | *PIK3CB* |
| *PIK3R1* | *PIM1* | *PMS2* | *POLD1* | *POLE* | *PPARG* | *PPP2R1A* | *PPP2R2A* | *PRDM1* | *PRKAR1A* |
| *PRKCI* | *PTCH1* | *PTEN* | *PTPN11* | *PTPRO* | *QKI* | *RAC1* | *RAD21* | *RAD51* | *RAD51B* |
| *RAD51C* | *RAD51D* | *RAD52* | *RAD54L* | *RAF1* | *RARA* | *RB1* | *RBM10* | *REL* | *RET* |
| *RICTOR* | *RNF43* | *ROS1* | *RPTOR* | *SDHA* | *SDHB* | *SDHC* | *SDHD* | *SETD2* | *SF3B1* |
| *SGK1* | *SMAD2* | *SMAD4* | *SMARC A4* | *SMARC B1* | *SMO* | *SNCAIP* | *SOCS1* | *SOX2* | *SOX9* |
| *SPEN* | *SPOP* | *SRC* | *STAG2* | *STAT3* | *STK11* | *SUFU* | *SYK* | *TBX3* | *TEK* |
| *TET2* | *TGFBR2* | *TIPARP* | *TNFAIP3* | *TNFRSF14* | *TP53* | *TSC1* | *TSC2* | *TYRO3* | *U2AF1* |
| *VEGFA* | *VHL* | *WHSC1* | *WHSC1L1* | *WT1* | *XPO1* | *XRCC2* | *ZNF217* | *ZNF703* |  |

B. Gene list: select rearrangements of the indicated genes were examined.

| *ALK intron 18, 19* | *BCL2 3′UTR* | *BCR intron 8, 13, 14* | *BRAF intron 7–10* | *BRCA1 intron 2, 7, 8, 12, 16, 19, 20* |
| --- | --- | --- | --- | --- |
| *BRCA2 intron 2* | *CD74 intron 6–8* | *EGFR intron 7, 15, 24–27* | *ETV4 intron 5, 6* | *ETV5 intron 6, 7* |
| *ETV6 intron 5, 6* | *EWSR1 intron 7–13* | *EZR intron 9–11* | *FGFR1 intron 1, 5, 17* | *FGFR2 intron 1, 17* |
| *FGFR3 intron 17* | *KIT intron 16* | *KMT2A(MLL) intron 6–11* | *MSH2 intron 5* | *MYB intron 14* |
| *MYC intron 1* | *NOTCH2 intron 26* | *NTRK1 intron 8–10* | *NTRK2 intron 12* | *NUTM1 intron 1* |
| *PDGFRA intron 7, 9, 11* | *RAF1 intron 4-8* | *RARA intron 2* | *RET intron 7–11* | *ROS1 intron 31-35* |
| *RSPO2 intron 1* | *SDC4 intron 2* | *SLC34A2 intron 4* | *TERC non-cording RNA* | *TERT promoter* |
| *TMPRSS2 intron 1–3* |  | | | |

**Table S2.** Success or failure with targeted next-generation sequencing of F1CDx for the 115 breast cancer samples in this study

A. Success and failure rate

|  | n | % |
| --- | --- | --- |
| Success | 109 | 94.8 |
| Failure | 6 | 5.2 |

B. Samples with failed results

| Sample No | Tumor type | Procedure | Site | Duration (days) | Previous therapy | Cellularity (%) | Tumor size | FMI Status |
| --- | --- | --- | --- | --- | --- | --- | --- | --- |
| 038 | Primary | CNB | Breast | 264 | - | 50 | 4 x 1 mm, 8 pieces | Failed testing |
| 044 | Metastatic | Resection | Breast | 1355 | CTx | 80 | 5 x 7 mm | Failed testing |
| 045 | Primary | Resection | Breast | 1423 | CTx | 50 | Scattered | Conditional TIFA |
| 053 | Primary | Resection | Breast | 347 | - | 70 | 17 x 23 mm | Failed testing |
| 094 | Metastatic | CT-guided biopsy | Lung | 19 | HTx | 40 | 2 x 3 mm, 2 x 1 mm | Conditional TIFA |
| 095 | Primary | CNB | Breast | 1085 | - | 30 | Scattered | Failed testing |

Duration (days): days from sample collection to next-generation sequencing examination

CNB, core needle biopsy; CT, computed tomography; CTx, chemotherapy; HTx, hormonal therapy; FMI, Foundation Medicine Inc.; TIFA, tissue insufficient for analysis

**Table S3.** Classification of potentially actionable alterations based on evidence

A. Clinical actionability according to OncoKB

| Level 1 | **FDA-recognized** biomarker predictive of response to an **FDA-approved drug** in this indication |
| --- | --- |
| Level 2 | **Standard care** biomarker recommended by the NCCN or other expert panels predictive of response to an **FDA-approved drug** in this indication |
| Level 3A | **Compelling clinical evidence** supports the biomarker as being predictive of response to a drug in this indication |
| Level 3B | **Standard care** or **investigational** biomarker predictive of response to an **FDA-approved** or **investigational** drug in another indication |
| Level 4 | **Compelling clinical evidence** supports the biomarker as being predictive of response to a drug in another indication |
| Level R1 | **Standard care** biomarker predictive of resistance to an **FDA-approved** drug in this indication |
| Level R2 | **Compelling clinical evidence** supports the biomarker as being predictive of **resistance** to a drug |

B. Clinical actionability according to the consensus of JCRSs and C-CAT

| JCRSs | C-CAT |  |
| --- | --- | --- |
| 1A | Level A | The biomarker predictive of response to a drug covered by Japanese health insurance in this indication |
| 1B |  | FDA-recognized biomarker predictive of response to an FDA-approved drug in this indication |
|  |  | Standard care biomarker predictive of response to a drug covered by Japanese health insurance in this indication |
| 2A | Level B | Compelling clinical evidence supports the biomarker as being predictive of response to a drug in this indication |
| 2B | Level C | Standard care biomarker predictive of response to a drug covered by Japanese health insurance in another indication, but not standard care in this indication |
|  |  | Compelling clinical evidence supports the biomarker as being predictive of response to a drug in another indication |
| 3A | Level D | Clinical evidence (case reports) supports the biomarker as being predictive of response to a drug |
| 3B | Level E | Compelling biological evidence supports the biomarker as being predictive of response to a drug |
| 4 | Level F | The biomarker is known to be involved in cancer |
|  | Level R | Resistance |

JCRSs, three major Japanese cancer-related societies; C-CAT, Center for Cancer Genomics and Advanced Therapeutics

C. Accessibility

| Level | Accessibility |
| --- | --- |
| 1 | Approved drugs |
| 2 | Clinical trial |
| 3 | Off-label use |
| 4 | Abroad clinical trial |
| 5 | FDA-approved drugs |
| 6 | None |

D. Therapies with clinical benefit according to genomic findings

| OncoKB EL | Gene | Alterations | Tumor type | Drugs | JCRSs/C-CAT EL | Accessibility |
| --- | --- | --- | --- | --- | --- | --- |
| 1 | Other biomarkers | MSI -high | All solid tumors | Pembrolizumab | A | 1 |
| 2B | Other biomarkers | MSI -high | Colon cancer | Nivolumab | C | 3 |
|  | Other biomarkers | MSI -high | Other tumor | Atezolizumab | D | 5 |
|  |  |  |  | Avelumab | C | 5 |
|  |  |  |  | Cemiplimab-rwlc | C | 5 |
|  |  |  |  | Durvalumab | C | 5 |
|  | Other biomarkers | TMB-high | Other tumor | Atezolizumab | C | 5 |
|  |  |  |  | Avelumab | C | 5 |
|  |  |  |  | Cemiplimab-rwlc | C | 6 |
|  |  |  |  | Durvalumab | C | 5 |
|  |  |  |  | Nivolumab | C | 5 |
|  |  |  |  | Pembrolizumab | C | 5 |
| 1 | ERBB2 | Amplification | Breast cancer | Ado-trastuzumab emtansine | A | 1 |
|  |  |  |  | Lapatinib | A | 1 |
|  |  |  |  | Neratinib | A | 5 |
|  |  |  |  | Pertuzumab | A | 1 |
|  |  |  |  | Trastuzumab | A | 1 |
|  | ERBB2 | Amplification | Breast cancer | Trastuzumab-dkst | A | 5 |
|  |  |  |  | Trastuzumab-dttb | A | 5 |
|  |  |  |  | Trastuzumab-pkrb | A | 5 |
|  |  |  |  | Trastuzumab-qyyp | A | 5 |
|  |  |  | Other tumor | Afatinib | C | 5 |
|  |  |  |  | Dacomitinib | C | 5 |
| 3A | ERBB2 | Oncogenic mutation | Breast cancer | Ado-trastuzumab emtansine | C | 5 |
| 3A | ERBB2 | Oncogenic mutation | Breast cancer | Neratinib | B | 5 |
|  | ERBB2 | Oncogenic mutation | Breast cancer | Lapatinib | C | 5 |
|  |  |  |  | Pertuzumab | C | 5 |
|  |  |  |  | Trastuzumab | C | 5 |
|  |  |  |  | Trastuzumab-dkst | C | 5 |
|  |  |  |  | Trastuzumab-dttb | C | 5 |
|  |  |  |  | Trastuzumab-pkrb | C | 5 |
|  |  |  |  | Trastuzumab-qyyp | C | 5 |
|  |  |  | NSCLSC | Afatinib | D | 5 |
|  |  |  | Other tumor | Dacomitinib | C | 5 |
| 2B | BRCA1/2 | Oncogenic mutation | Ovarian cancer, Peritoneal serous carcinoma | Niraparib | C | 3 |
| 2A | BRCA1/2 | Oncogenic mutation | Breast cancer | Olaparib | B | 1 |
| 2B | BRCA1/2 | Oncogenic mutation | Ovarian cancer, Peritoneal serous carcinoma | Rucaparib | C | 3 |
| 2A | BRCA1/2 | Oncogenic mutation | Breast cancer | Talazoparib | B | 3 |
| 3A | AKT1 | E17K | Breast cancer | ADZ5363 | C | 2 |
|  |  | Oncogenic mutation | Breast cancer | Everolimus | C | 5 |
|  |  |  | Other tumor | Temsirolimus | C | 5 |
|  | AKT3 | Oncogenic mutation | Breast cancer | Everolimus | C | 5 |
|  |  |  | Other tumor | Temsirolimus | C | 5 |
|  | AR | Amplification | Other tumor | Abiraterone | C | 6 |
|  |  |  |  | Bicalutamide | C | 6 |
|  |  |  |  | Degarelix | C | 6 |
|  |  |  |  | Flutamide | C | 6 |
|  |  |  | Breast cancer | Goserelin | C | 5 |
|  |  |  | Other tumor | Leuprolide | C | 5 |
|  |  |  |  | Nilutamide | C | 6 |
|  |  |  |  | Triptorelin | C | 6 |
| 4A | ATM | Oncogenic mutation | All solid tumors | Olaparib | E | 6 |
|  |  |  | Other tumor | Niraparib | E | 6 |
|  |  |  |  | Rucaparib | E | 6 |
|  |  |  |  | Talazoparib | E | 2 |
| 3B | BRAF | Fusion | Ovarian Cancer, Melanoma | Cobimetinib, Trametinib | C | 3 |
|  |  | Oncogenic mutation | Histocytosis | Cobimetinib | C | 3 |
| 2B | BRAF | V600E | Melanoma | Dabrafenib | C | 3 |
|  |  |  |  | Vemurafenib | C | 3 |
| 2B | BRAF | V600E, V600K | Melanoma | Binimetinib+Encorafenib | C | 3 |
|  |  |  |  | Cobimetinib+Vemurafenib | C | 3 |
|  |  |  |  | Trametinib | C | 3 |
|  | BRAF | V600E | Other tumor | Regorafenib | D | 6 |
|  | BRAF | Fusions | Other tumor | Sorafenib | D | 6 |
|  | CCND1 | Amplification | Breast cancer | Abemaciclib | C | 6 |
|  |  |  | Other tumor | Palbociclib | C | 6 |
|  |  |  |  | Ribociclib | C | 6 |
|  | CD274(PD-L1) | Amplification | Breast cancer | Atezolizumab | E | 6 |
|  |  |  | Other tumor | Avelumab | E | 6 |
|  |  |  |  | Cemiplimab-rwlc | E | 6 |
|  |  |  |  | Durvalumab | E | 6 |
|  |  |  |  | Nivolumab | E | 6 |
|  |  |  |  | Pembrolizumab | E | 3 |
|  | CDK4 | Amplification or activation | Breast cancer | Ribociclib | C | 6 |
| 2B | CDK4 | Amplification | Liposarcoma | Abemaciclib | C | 6 |
|  |  |  |  | Palbociclib | C | 6 |
| 4A | CDK12 | Truncating mutations | All solid tumors | Cemiplimab, Nivolumab, Pembrolizumab | E | 6 |
| 4A | CDKN2A | Oncogenic mutations | All solid tumors | Abemaciclib, Palbociclib, Ribociclib | E | 6 |
|  | ERBB3 | Oncogenic mutations | Breast cancer | Ado-trastuzumab emtansine | C | 6 |
|  |  |  | Other tumor | Afatinib | C | 6 |
|  |  |  | Breast cancer | Lapatinib | C | 6 |
|  |  |  |  | Neratinib | C | 6 |
|  |  |  |  | Pertuzumab | C | 6 |
|  |  |  |  | Trastuzumab | C | 6 |
|  |  |  |  | Trastuzumab-dkst | C | 6 |
|  |  |  |  | Trastuzumab-dttb | C | 6 |
|  |  |  |  | Trastuzumab-pkrb | C | 6 |
|  |  |  |  | Trastuzumab-qyyp | C | 6 |
| 3A | ESR1 | Oncogenic mutations | Breast cancer | Fulvestrant | C | 6 |
| R | ESR1 | Oncogenic mutations |  | Anastrozole | R |  |
|  |  |  |  | Exemestane | R |  |
|  |  |  |  | Letrozole | R |  |
| 3B | EZH2 | Oncogenic mutations | Follicular lymphoma | Tazemetostat | C | 5 |
|  | FBXW7 | Loss or inactivation | Other tumor | Everolimus | C | 6 |
|  |  |  |  | Temsirolimus | C | 6 |
|  | FGFR1/2 | Amplification | Other tumor | Pazopanib | D | 6 |
|  |  |  |  | Ponatinib | E | 6 |
| 2B | FGFR2 | Fusions | Bladder cancer | Erdafitinib | C | 2 |
| 3B | FGFR1 | Amplification | Lung squamous cell carcinoma | AZD4547, BGJ398, Debio1347, Erdafitinib | C | 6 |
| 3B | FGFR2 | Fusions | Bladder cancer | AZD4547, BGJ398, Debio1347 | C | 6 |
|  |  |  | Cholangiocarcinoma | AZD4547, BGJ398, Debio1347, Erdafitinib | C | 6 |
| 4A | FGFR1 | Oncogenic mutations | All solid tumors | AZD4547, BGJ398, Debio1347, Erdafitinib | E | 6 |
| 4A | FGFR2 | Oncogenic mutations | All solid tumors | AZD4547, BGJ398, Debio1347, Erdafitinib | E | 6 |
| 3B | HRAS | Oncogenic mutations | Head and neck squamous cell carcinoma | Tipifarnib | C | 4 |
|  |  | Activating mutations | Other tumor | Binimetinib | E | 6 |
|  |  |  |  | Cobimetinib | E | 6 |
|  |  |  |  | Trametinib | E | 6 |
|  | KDR | Activating mutations | Other tumor | Sunitinib | E | 6 |
| 4A | KRAS | Oncogenic mutations | All solid tumors | Binimetinib | E | 6 |
|  |  |  |  | Cobimetinib | E | 6 |
|  |  |  |  | Trametinib | E | 6 |
| 3B | KRAS | Oncogenic Mutations | Histocytosis | Cobimetinib | C | 6 |
| 3B | MDM2 | Amplification | Liposarcoma | Milademetan Tosylate, RO5045337 | C | 6 |
| 4A | NF1 | Oncogenic mutations | All solid tumors | Cobimetinib | E | 6 |
| 4A | NF1 | Oncogenic mutations | All solid tumors | Trametinib | E | 6 |
|  | NF1 | Inactivation | Other tumor | Binimetinib | E | 6 |
|  | NF2 | Inactivation | Other tumor | Everolimus | E | 6 |
|  |  |  |  | Temsirolimus | E | 6 |
|  | PDCD1LG2(PD-L2) | Amplification | Other tumor | Atezolizumab | E | 6 |
|  |  |  |  | Avelumab | E | 6 |
|  |  |  |  | Cemiplimab-rwlc | E | 6 |
|  |  |  |  | Durvalumab | E | 6 |
|  |  |  |  | Nivolumab | E | 6 |
|  |  |  |  | Pembrolizumab | E | 6 |
|  | PDGFRA | Activating mutations | Other tumor | Sorafenib | C | 6 |
| 2B | PDGFRA | D842V | Gastrointestinal stromal tumor | Dasatinib | C | 6 |
| 2B | PDGFRA | Oncogenic mutations | Gastrointestinal stromal tumor | Imatinib | C | 6 |
| 1 | PIK3CA | Oncogenic mutations | Breast cancer | Alpelisib + Fulvestrant | B | 2 |
| 3A | PIK3CA | Oncogenic mutations | Breast cancer | Alpelisib | A | 2 |
|  |  |  |  | Buparlisib | B | 5 |
|  |  |  |  | Buparlisib + Fulvestrant | B | 5 |
|  |  |  |  | Copanlisib | B | 5 |
|  |  |  |  | Fulvestrant + Taselisib | B | 5 |
|  |  |  |  | GDC-0077 | B | 6 |
|  |  |  |  | Serabelisib | B | 6 |
|  |  |  |  | Taselisib | B | 5 |
|  | PIK3CA | Oncogenic mutations | Breast cancer | Everolimus | C | 6 |
|  |  |  | Other tumor | Temsirolimus | C | 6 |
| 3B | PTCH1 | Truncating mutations | Skin cancer, Non-melanoma embryonal tumor | Sonidegib | C | 6 |
|  |  |  | Skin cancer, Non-melanoma | Vismodegib | C | 6 |
|  | PTEN | Loss or inactivation | Breast cancer | Everolimus | C | 6 |
|  |  |  | Other tumor | Temsirolimus | C | 6 |
| 4A | PTEN | Oncogenic Mutations | All solid tumors | AZD8186, GSK2636771, ADZ5363 | E | 2 |
|  | RAF1 | Amplification or activation | Other tumor | Regorafenib | C | 6 |
|  |  |  | Other tumor | Sorafenib | C | 6 |
| 3B | RAF1 | Oncogenic mutations | Histiocytosis | Cobimetinib | C | 6 |
|  | STK11 | Oncogenic mutations | Breast cancer | Everolimus | D | 6 |
|  |  |  | Other tumor | Temsirolimus | D | 6 |
|  | TSC1 | Oncogenic mutations | Other tumor | Temsirolimus | C | 6 |
| 2B | TSC1 | Oncogenic mutations | CNS cancer | Everolimus | C | 6 |
| 2B | TSC1 | Oncogenic mutations | Renal cell carcinoma | Everolimus | C | 6 |
|  | TSC1 | Oncogenic mutations | Breast cancer | Everolimus | C | 6 |
| 4 | SF3B1 | Oncogenic mutations | Chronic myelomonocytic leukemia | H3B-8800 | E | 6 |

OncoKB EL, evidence level according to OncoKB; JCRSs/C-CAT EL, evidence level according to the consensus of JCRSs and C-CAT
